# FSCA-YOLO: An Enhanced YOLO-Based Model for Multi-Target Dairy Cow Behavior Recognition

**DOI:** 10.3390/ani15172631

**Published:** 2025-09-08

**Authors:** Ting Long, Rongchuan Yu, Xu You, Weizheng Shen, Xiaoli Wei, Zhixin Gu

**Affiliations:** 1 College of Computer and Control Engineering, Northeast Forestry University, Harbin 150040, China; lttx@nefu.edu.cn (T.L.); yurongchuan@nefu.edu.cn (R.Y.); 2874095847@nefu.edu.cn (X.Y.); 2 College of Electric and Information, Northeast Agricultural University, Harbin 150030, China; wzshen@neau.edu.cn

**Keywords:** cow behavior, multi-object detection, behavior recognition, YOLOv11

## Abstract

In real-world dairy farming environments, object recognition models often suffer from missed or false detections due to complex backgrounds and cow occlusions. In response to these issues, this paper proposes FSCA-YOLO, a multi-object cow behavior recognition model based on an improved YOLOv11 framework. First, the FEM-SCAM module is introduced along with the CoordAtt mechanism to enable the model to better focus on effective behavioral features of cows while suppressing irrelevant background information. Second, a small object detection head is added to enhance the model’s ability to recognize cow behaviors occurring at the distant regions of the camera’s field of view. Finally, the original loss function is replaced with the SIoU loss function to improve recognition accuracy and accelerate model convergence. Experimental results show that compared with mainstream object detection models, the improved YOLOv11 in this section demonstrates superior performance in terms of precision, recall, and mean average precision (mAP), achieving 95.7% precision, 92.1% recall, and 94.5% mAP—an improvement of 1.6%, 1.8%, and 2.1%, respectively, over the baseline YOLOv11 model. FSCA-YOLO can accurately extract cow features in real farming environments, providing a reliable vision-based solution for cow behavior recognition. To support specific behavior recognition and in-region counting needs in multi-object cow behavior recognition and tracking systems, OpenCV is integrated with the recognition model, enabling users to meet the diverse behavior identification requirements in groups of cows and improving the model’s adaptability and practical utility.

## Simple Summary

In precision dairy farming, accurately recognizing cow behaviors is essential for health monitoring and herd management. However, complex barn environments and high inter-cow similarity pose challenges for behavior recognition systems. To address these issues, we collected real-world video data from both indoor and outdoor areas of a dairy farm and proposed an enhanced multi-behavior recognition model based on YOLOv11 (You Only Look Once, version 11). By integrating a Feature Enhancement Module and attention mechanisms, the model effectively improves feature extraction in complex scenes. Experimental results demonstrate that our method achieves superior accuracy in detecting feeding, drinking, standing, and lying behaviors in groups of cows, offering a reliable and efficient tool for intelligent dairy farm management.

## 1. Introduction

The daily health and behavior recognition of dairy cows is a crucial task in farm management. One of the primary methods for identifying the health status of dairy cows is observing their behavior, as abnormal behaviors often indicate potential issues with their mental or physical condition [1,2]. With the digital transformation of the livestock industry [3], the massive and diverse data generated by farming operations has become the norm [4,5,6]. However, the human brain is incapable of storing and processing such complex data. The accuracy of this data directly impacts the scientific basis of decision-making and the consistency of farming quality [7,8].

With the rapid development of information technology, it has become possible to automatically and continuously recognize cattle behavior, mainly through contact-based and non-contact-based methods [2]. In recent years, the development and decreasing cost of various sensor technologies have greatly facilitated the advancement of behavior recognition [9]. Displacement sensors, also known as linear sensors, are linear devices based on metal sensing. Their working principle involves converting various physical measurements into electrical signals [10]. These signals can be easily transmitted and analyzed, enabling the monitoring and quantification of the measured variables [11]. Wearable systems—including a triaxial-accelerometer neck collar that aligns strongly with visual labels (feeding r = 0.91, rumination r = 0.89; high concordance correlation coefficient (CCC) and coefficient of determination (R^2^)) [12,13], the multisensor RumiWatch (noseband pressure + leg accelerometer) [14], and three-axis accelerometer-based classifiers [15]—have demonstrated the accurate monitoring of key bovine behaviors (feeding, rumination, drinking, and locomotion) across pasture and confined settings. However, because sensors are worn on the animals, they can fall off easily and may cause discomfort, affecting animal welfare. As a result, non-contact computer vision-based methods have become a popular trend for behavior recognition [16,17,18].

As a non-contact and stress-free method, computer vision technology can recognize basic cow behaviors with higher precision and efficiency [19,20]. In complex farm settings, Wu et al. [21] employed a VGG16-BiLSTM framework to recognize five key behaviors with high accuracy (average precision 0.971, accuracy 0.976). Lodkaew [22] developed CowXNet based on YOLOv4 for estrus detection, achieving 83% accuracy. Guo et al. [23] applied background subtraction and frame differencing to monitor calf–environment interactions with 94.4% accuracy. Wang et al. [24] proposed an improved YOLOv8n (E-YOLO), which achieved 93.9% accuracy in estrus behavior detection. However, most of these methods either target specific behaviors or show reduced robustness under complex farm conditions, such as occlusion, varying illumination, and dense group interactions. These challenges necessitate more advanced approaches for accurate and scalable behavior monitoring.

In recent years, deep learning-based approaches have been increasingly applied to livestock behavior monitoring [25,26]. For instance, Li et al. [27] constructed a dataset covering nine typical beef cattle behaviors (standing, lying, mounting, fighting, licking, feeding, drinking, walking, and searching) under diverse conditions such as varying illumination and group densities. They proposed the YOLOv8n-BiF-DSC model, which integrates Dynamic Snake Convolution (DSC) to expand the receptive field and BiFormer attention to enhance long-range context modeling. Their method achieved superior recognition performance, with an accuracy of 93.6% and an mAP@0.5 of 96.5%, representing significant improvements of 5.3%, 5.2%, and 7.1% over the original YOLOv8n in accuracy, mAP@0.5, and mAP@0.5:0.95, respectively. Building upon this, Li et al. [28] further extended behavior recognition to multi-object identification and tracking. By embedding the improved YOLOv8n-BiF-DSC into the Deep SORT framework and optimizing trajectory association strategies—such as adopting ResNet18 for re-identification and incorporating a secondary Intersection over Union (IoU) matching scheme—they developed a non-invasive framework for behavior identification and tracking. Experimental results showed a 65.8% reduction in ID switches and a 2% increase in Multiple Object Tracking Accuracy (MOTA), highlighting the robustness and reliability of the framework for group-housed cattle monitoring.

It is evident that deep learning models are now widely applied in dairy cow behavior recognition [29,30]. However, based on the reviewed studies, there is still room for expanding the behavioral types and data acquisition areas [31,32]. Additionally, feature extraction capabilities of the models need further improvement to cope with the complex and variable farm environments [33]. Under real-world farm conditions, cow behavior recognition faces several challenges: cows often overlap at narrow feed troughs, where torsos and heads are blocked by other cows or railings [34]; dense groups frequently crowd around water troughs, causing severe target overlap; and lying cows are partially hidden by bed structures, with infrared imaging at night further lowering contrast. In addition, illumination changes and cluttered backgrounds make detection more difficult [35]. Therefore, collecting behavioral data from multiple regions to enrich behavior datasets and modifying recognition models to enhance their performance remain pressing research needs. This study aims to improve the feature extraction capabilities of the initial YOLOv11 [36] model through several enhancements:

- Replacing the backbone module with the Feature Enhancement Module-Spatial Context Awareness Module (FEM-SCAM) module, integrating the Coordinate Attention (CoordAtt) mechanism [37] to improve the expression of image features [38].
- Adding a small-object detection head to enhance the recognition of tiny dairy cow targets [39].
- replacing the Structured Intersection over Union  (SIoU) loss function [40] to optimize the alignment between predicted and actual targets [30,40].
- To support downstream applications such as behavior-specific cow counting and tracking in defined zones, the model was integrated with Open Source Computer Vision Library (OpenCV)-based tools [17,41,42]. This integration broadens the applicability of the system and promotes the development of the emerging paradigm of the “digital dairy farm”.

Ablation experiments and performance comparisons with other target detection algorithms are conducted to validate that the proposed model achieves superior detection performance for dairy cow behaviors in complex environments. Furthermore, visualization results demonstrate that the model maintains high robustness under challenges such as occlusion and background interference, highlighting its effectiveness in practical scenarios. In addition, the proposed framework can be seamlessly integrated with OpenCV-based tools for region-specific counting and tracking, which further confirms its applicability and ease of deployment in real-world farm management.

## 2. Materials and Methods

### 2.1. Dataset Preparation

#### 2.1.1. Data Acquisition

**Figure 1:**
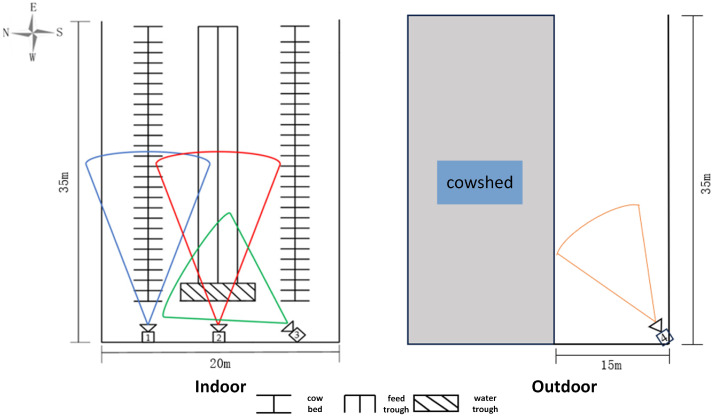
Schematic diagram of indoor and outdoor activity area image acquisition in a dairy farm.

The video data used in this study were collected at the Shengkang Livestock Farm in Daqing, Heilongjiang Province, China. Dairy cows are fed twice daily, from 5:00 to 6:00 in the morning and from 17:00 to 18:00 in the evening. Therefore, video clips capturing feeding behavior were collected during these two time intervals. Drinking and standing behaviors primarily occur during the daytime when lighting conditions are sufficient, and the corresponding data were also collected during this period. The barn’s surveillance system switches to night vision mode at 19:00 each day. Accordingly, the nighttime lying behavior was extracted from surveillance footage recorded between 19:00 and 3:00 the following morning. Additional lying behavior data were collected during daylight hours in the outdoor activity area. The dairy cow activity area was divided into an indoor barn area and an outdoor activity area as illustrated in Figure 1. The indoor area was equipped with feed troughs, water troughs, walking lanes, and cow bedding stalls. Four 4-megapixel surveillance cameras (Hangzhou Hikvision Digital Technology Co., Ltd., Hangzhou, China, model DS-IPC-B14H-LFT) were deployed to capture cow behavior videos.

- Camera 1, indicated in blue, was positioned to monitor the walking lane and partially covered the bedding area, making it suitable for recording both the standing behavior during the day and infrared images of the lying behavior at night.
- Camera 2, marked in red, focused on the feeding area, and was used primarily to capture the feeding behavior.
- Camera 3, marked in green, centered on the water trough and was used to collect data on the drinking behavior.
- Camera 4, indicated in orange, monitored the outdoor area and was used to capture both the daytime lying behavior and the standing behavior outside the barn.

Cameras 1, 2, and 4 were installed at a $30^{\circ }$ angle relative to the horizontal plane, while Camera 3 was installed at a $45^{\circ }$ angle.

#### 2.1.2. Data Preprocessing

**Figure 2:**
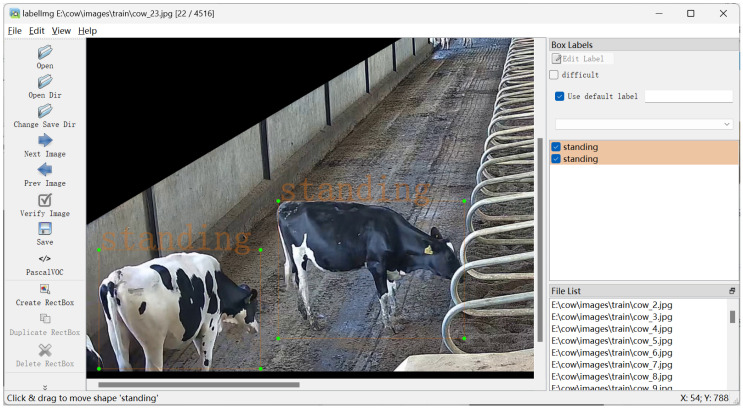
Labelimg Software interface (version 1.8.6).

**Figure 3:**
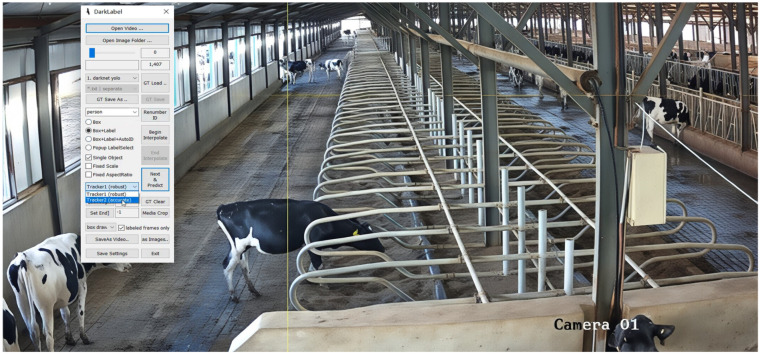
Darklabel software interface (version 2.4).

To obtain a comprehensive and high-quality dataset of dairy cow behaviors, the initial surveillance videos collected from the dairy farm were subjected to a screening and segmentation process. A total of 35 indoor monitoring videos were collected, each with a duration of one hour. First, videos were manually by a person reviewed to eliminate segments with issues such as overexposure, lens contamination, motion blur due to cow movement, or the absence of cows within the region of interest. Then, Python (version 3.13.1) scripts using the MoviePy library were developed to segment the qualified videos into 2-min clips. Finally, the OpenCV computer vision library was used to extract image frames at 2-s intervals from each video clip. In this study, cow behavior was annotated using the LabelImg software (version 1.8.6), with its interface shown in Figure 2. DarkLabel (version 2.4) was used as an auxiliary tool for dataset annotation to ensure high-quality labeling for the behavior-recognition task as illustrated in Figure 3. LabelImg (https://github.com/heartexlabs/labelImg (accessed on 6 January 2025)) was used for behavior annotation, and DarkLabel (https://github.com/darkpgmr/DarkLabel (accessed on 6 January 2025) was used as an auxiliary tool).

**Figure 4:**
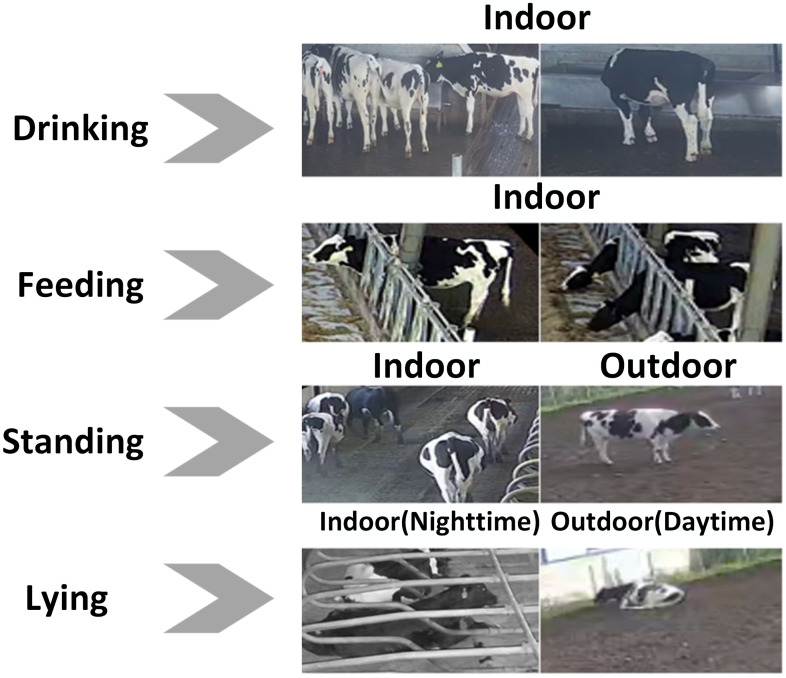
Images of various cow behaviors.

The annotation criteria for each cow behavior were as follows, with representative images shown in Figure 4.

1. For feeding behavior, when cows feed side by side and occlusion occurs, targets with more than 50% occlusion or less than 15% visible area near the image edge were not labeled.
2. For lying behavior, due to the unique coat patterns of dairy cows, some lying cows with fully black backs that closely resemble the background were excluded from annotation. This exclusion accounts for 3.2% (12/372) of lying candidates, where 12 denotes excluded cows with completely black backs that were visually indistinguishable from the background, and 372 is the total number of lying candidates (excluded + labeled instances).
3. For standing behavior, only cows whose four legs were in contact with the ground or whose legs were naturally bent during movement were labeled.
4. For drinking behavior, as such actions occur only near the water trough, only targets where the cow’s head entered the trough area were annotated.

In total, 3360 images were collected for model training and evaluation. Considering the stability of lying behavior at night, 360 images were selected for this category, while 1000 images were collected for each of the other three behaviors (standing, eating, and drinking). This distribution inevitably introduced class imbalance into the dataset. To address this issue and enhance model robustness, we employed the Mosaic augmentation strategy integrated in YOLOv11 during training. Mosaic combines four training images into one, which effectively enriches object contexts, increases the diversity of object scales and positions, and improves the model’s generalization ability. This augmentation mitigates the negative impact of class imbalance and contributes to more reliable behavior recognition results.

### 2.2. Network Structure of FSCA-YOLO

#### 2.2.1. YOLOv11 Backbone Network

**Figure 5:**
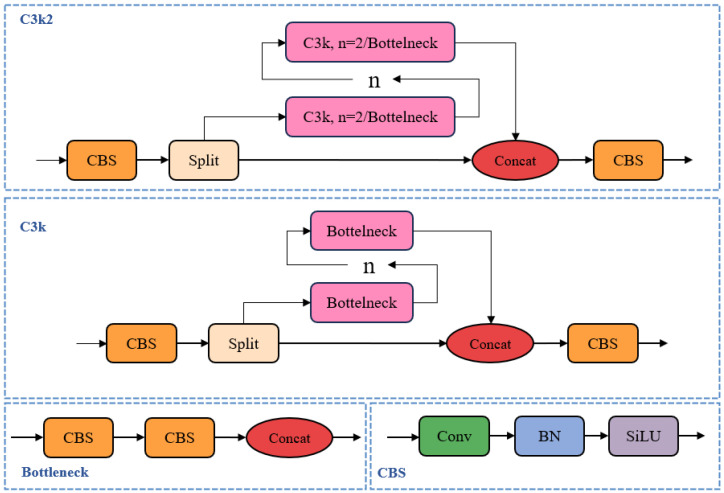
The C3k2 structure diagram.

**Figure 6:**
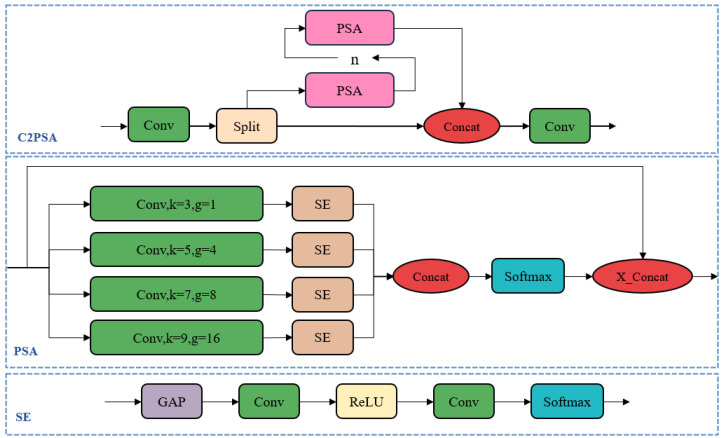
The C2PSA structure diagram.

YOLOv11 inherits the efficient design philosophy characteristic of the YOLO series and achieves significant improvements in terms of accuracy, speed, and deployability [43,44]. Compared to earlier YOLO models, YOLOv11 introduces numerous architectural optimizations, with the main improvements as follows:

(1) C3k2 module. YOLOv11 retains the core design concept of Cross Stage Partial (CSP) from CSPDarkNet, while further optimizing its hierarchical structure and channel configuration. The C3k2 module, a key component of the YOLOv11 backbone, combines standard and grouped convolutions and supports flexible configuration using either the C3k module with custom kernel sizes or a standard bottleneck. This design enhances feature extraction efficiency while adapting to various computational constraints as illustrated in Figure 5.

(2) C2PSA Module. The C2PSA module is a convolutional block based on the PSA (Pyramid Squeeze Attention) attention mechanism [45], designed to process input tensors and enhance feature representation through attention. This module involves convolution operations, feature splitting, and a multi-head attention mechanism. The network structure is illustrated in Figure 6.

The SE (Squeeze-and-Excitation) module performs dynamic weighting based on channel features. First, it applies Global Average Pooling (GAP) across the channel dimension. Then, it extracts channel-wise weights using a 1 × 1 convolution followed by a Rectified Linear Unit (ReLU) activation function. Finally, the channel weights are obtained through a Softmax function. This module enhances the responses of important channels while suppressing less relevant ones, thereby improving the model’s ability to capture critical features. Similarly, the PSA module adopts the concept of multi-scale convolutional kernels, extracting features in parallel using convolutional kernels of different sizes. This allows the model to capture feature information of objects at varying scales. The C2PSA module concatenates the feature information extracted by multiple PSA modules with the original feature information, thereby enhancing the model’s ability to capture multi-scale features.

#### 2.2.2. FEM-SCAM Integration

**Figure 7:**
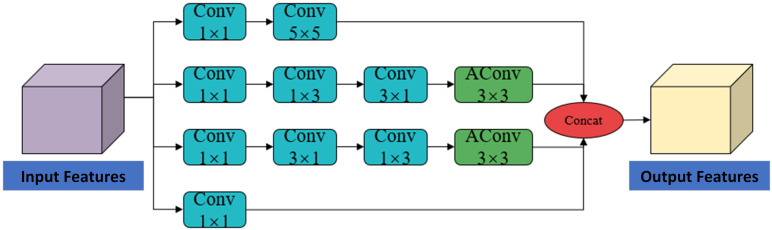
FEM module architecture diagram.

In real farming environments, irrelevant objects and visual similarities between cows and the background often cause missed or false detections. To address this, we propose a Feature Enhancement Module (FEM) based on the C3k2 structure for improved contextual feature extraction, and a Spatial Context Awareness Module (SCAM) to suppress background noise and enhance target discrimination. This section proposes FEM that utilizes multi-branch convolution and dilated convolution to enrich feature representation and expand the receptive field, replacing the original bottleneck structure in the C3k2 module. The structure of the FEM module is shown in Figure 7. It consists of four branches, each containing a 1 × 1 convolutional layer to adjust the number of channels. The first branch employs a 5 × 5 large kernel convolution, allowing the model to progressively expand the receptive field while maintaining the spatial resolution of the feature map, thereby enabling it to capture broader contextual information. This contributes to a better understanding and recognition of the overall behavioral features of cows. The second and third branches extract features using 1 × 3 and 3 × 1 convolutional kernels in different orders, allowing the model to acquire information from the horizontal and vertical directions, respectively. Both branches incorporate dilated convolutions to further preserve the contextual information in the feature maps. The final branch retains the original features through a residual connection and integrates them with the outputs of the other three branches.

**Figure 8:**
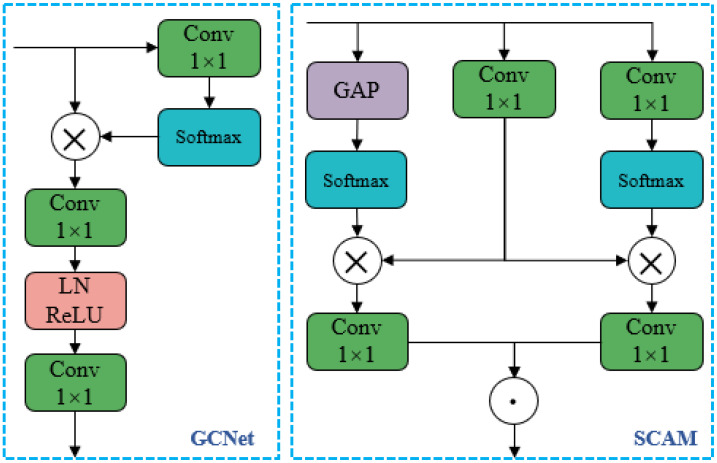
Structure diagram of GCNet and SCAM modules.

GCNet [46] (Global Context Network) is a neural network architecture designed for global context modeling, aiming to more effectively capture long-range dependencies and enhance performance in visual recognition tasks. The network structure is illustrated in Figure 8. First, the input features are passed through a 1 × 1 convolution kernel and a Softmax function to obtain attention weights, which are then used to derive global context features. Next, the global context features are multiplied with the original feature matrix. Finally, a bottleneck structure composed of two 1 × 1 convolution kernels, Layer Normalization (LN), and the ReLU activation function is used to capture inter-channel dependencies and reduce parameter redundancy. Based on this structure, this section introduces improvements to the feature transformation mechanism and context information fusion by incorporating a spatial context-aware module.

The SCAM module consists of three branches. The first branch employs Global Average Pooling (GAP), which effectively integrates global feature information of the image and captures long-range dependencies within it. The second branch applies a 1 × 1 convolution to generate a linear transformation of the feature maps, enabling the adjustment of feature dimensions and distributions. This enhances the model’s capacity to represent diverse features, allowing it to better adapt to regional variations and improve feature quality and discriminability. The third branch also uses a 1 × 1 convolution to simplify the association between image regions and features. Depending on the extraction positions, the model performs a weighted operation on the feature values to extract more representative information, eliminate redundancy and noise, and further refine the features, thereby enhancing the model’s focus on critical information. Finally, the outputs from the first and third branches are each matrix-multiplied with that of the second branch. The resulting matrices are passed through another 1 × 1 convolution to unify feature dimensions. These two outputs respectively represent the contextual information across channels and in spatial domains. The final step fuses these two types of features using Hadamard (element-wise) multiplication.

#### 2.2.3. P2 Head Addition

**Figure 9:**
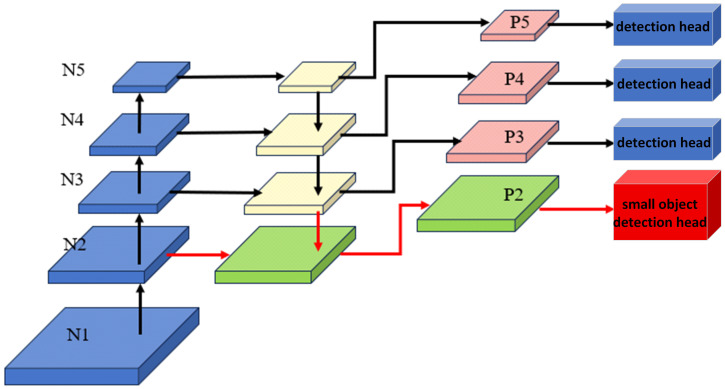
Improved model recognition structure schematic.

During the analysis of the cow image dataset, it was observed that due to the rectangular layout of cow farms, cows located at the far end of the camera’s field of view often occupy only a small portion of the overall image. After passing through multiple layers of feature extraction, small-scale cow behavior targets may lose part of their feature information, which ultimately affects the accuracy of behavior recognition. As shown in Figure 9, layers N1 to N5 represent the feature extraction stages in the original YOLOv11 model. Considering both the training efficiency and the retention of fine-grained details in the original images, the input image size in this study is set to 640 × 640. After downsampling by the network, the original model’s largest feature map size available for detection is 80 × 80, and the smallest is 20 × 20. For recognizing cow behavior at the far end of the camera view, additional feature information is required to improve recognition accuracy. Therefore, following the multi-scale detection design of the YOLO family [47,48], this study introduces an additional detection head, P2, on a higher-resolution feature map (160 × 160) to enhance small object detection. The P2 head has been widely adopted in later open-source YOLO implementations (e.g., YOLOv5/YOLOv8), although it has not been formally introduced in the original YOLO papers. The added P2 layer can more effectively extract complete and useful features from the lower-level network, and it helps mitigate issues such as false detections, missed detections, and low confidence scores.

#### 2.2.4. CoordAtt Integration

**Figure 10:**
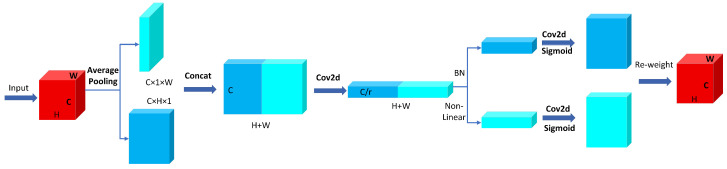
Coordinate attention mechanism.

To enhance the model’s ability to represent features of different dairy cow behaviors, this study incorporates the CoordAtt [37] mechanism into the backbone of YOLOv11 as shown in Figure 10, which encodes both horizontal and vertical positional information into the channel attention. This enables the network to capture long-range positional dependencies while introducing minimal computational overhead, thereby reducing the overall computation and enhancing the robustness of the model. The computation of the CoordAtt mechanism mainly consists of three steps:

1. Information Decomposition. For an input feature map of size *C* × *H* × *W*, Global Average Pooling is applied separately along the horizontal and vertical directions, compressing the 2D spatial information into 1D vectors. This results in a horizontal feature map of size *C* × *H* × 1 and a vertical feature map of size *C* × 1 × *W*. This decomposition effectively captures directional information in the feature map and prepares for subsequent positional encoding.
2. Feature Transformation. The horizontal and vertical features are concatenated along the spatial dimension to form a feature of size *C* × (*H* + *W*) × 1, which is then processed through a 1 × 1 convolution (Conv2d) followed by an activation function. After that, the feature is split along the spatial dimension into two separate tensors. Each tensor is then passed through another convolution operation followed by a Sigmoid activation function, producing attention vectors for the horizontal and vertical directions, respectively.
3. Reweighting. The attention vectors obtained in the second step are broadcasted to the original feature map size *C* × *H* × *W*. These vectors are then multiplied element-wise with the original input feature map to produce the final attention-enhanced features.

#### 2.2.5. Improved SIoU Loss Function

In the early development of multi-object detection, IoU [49] was widely adopted to evaluate model performance by quantifying the overlap between predicted and ground-truth bounding boxes. To address some limitations of IoU, GIoU [50] introduced penalties based on the enclosing box, Distance-IoU (DIoU) [51] focused on minimizing the center distance, and Complete Intersection over Union  (CIoU) [52] further integrated aspect ratio consistency. Despite these improvements, they do not consider the directional alignment between predicted and target boxes, which can slow convergence and degrade performance due to random directional shifts during training. To overcome this, the SIoU loss function [40] is adopted in this study. SIoU enhances regression by incorporating angle information between bounding box vectors, guiding predictions to align with the target box axis. This directional constraint reduces redundant movements and enables more efficient single-axis optimization. The SIoU loss comprises four components: angle loss, distance loss, shape loss, and IoU loss.

The following equation defines the angle loss: $c_{h}$ represents the height difference between the center point in the ground-truth coordinate system and that in the predicted coordinate system, while σ denotes the Euclidean distance between these two center points. $b_{c_{x}}^{gt}$, $b_{c_{y}}^{gt}$ indicates the center coordinates of the ground-truth object, whereas $b_{c_{x}}$, $b_{c_{y}}$ represents the center coordinates of the predicted bounding box generated by the model:

(1) $$\Lambda =1-2\cdot \sin^{2}\left(\arcsin\left(\frac{c_{h}}{\sigma }\right)-\frac{\pi }{4}\right)$$

(2) $$\sigma =\sqrt{{\left(b_{c_{x}}^{gt}-b_{c_{x}}\right)}^{2}+{\left(b_{c_{y}}^{gt}-b_{c_{y}}\right)}^{2}}$$

(3) $$c_{h}=\max\left(b_{c_{x}}^{gt}-b_{c_{y}}\right)-\min\left(b_{c_{x}}^{gt}-b_{c_{y}}\right)$$

The following equation defines the distance loss function, where $c_{w}$ and $c_{h}$ denote the width and height of the minimum enclosing rectangles of the ground-truth box and the predicted bounding box, respectively:

(4) $$\Delta =2-e^{-\gamma \rho_{x}}-e^{-\gamma \rho_{y}}$$

(5) $$\rho_{x}={\left(\frac{b_{c_{x}}^{gt}-b_{c_{x}}}{c_{w}}\right)}^{2},\rho_{y}={\left(\frac{b_{c_{y}}^{gt}-b_{c_{y}}}{c_{h}}\right)}^{2}$$

(6) γ=2−Λ#

The following equation defines the shape loss: *w* and *h* represent the width and height of the predicted bounding box and the ground-truth box, respectively. θ is a tunable parameter that indicates the weight assigned by the network to the shape loss:

(7) $$\Omega ={\left(1-e^{-\omega_{w}}\right)}^{\theta }+{\left(1-e^{-\omega_{h}}\right)}^{\theta }$$

(8) $$\omega_{w}=\frac{|w-w^{gt}|}{\max(w,w^{gt})},\;\omega_{h}=\frac{|h-h^{gt}|}{\max(h,h^{gt})}$$

The following equation defines the IoU loss function:

(9) $$Loss=1-IoU+\frac{\Delta +\Omega }{2}$$

(10) $$IoU=\frac{A\cap B}{A\cup B}$$

#### 2.2.6. FSCA-YOLO Model

**Figure 11:**
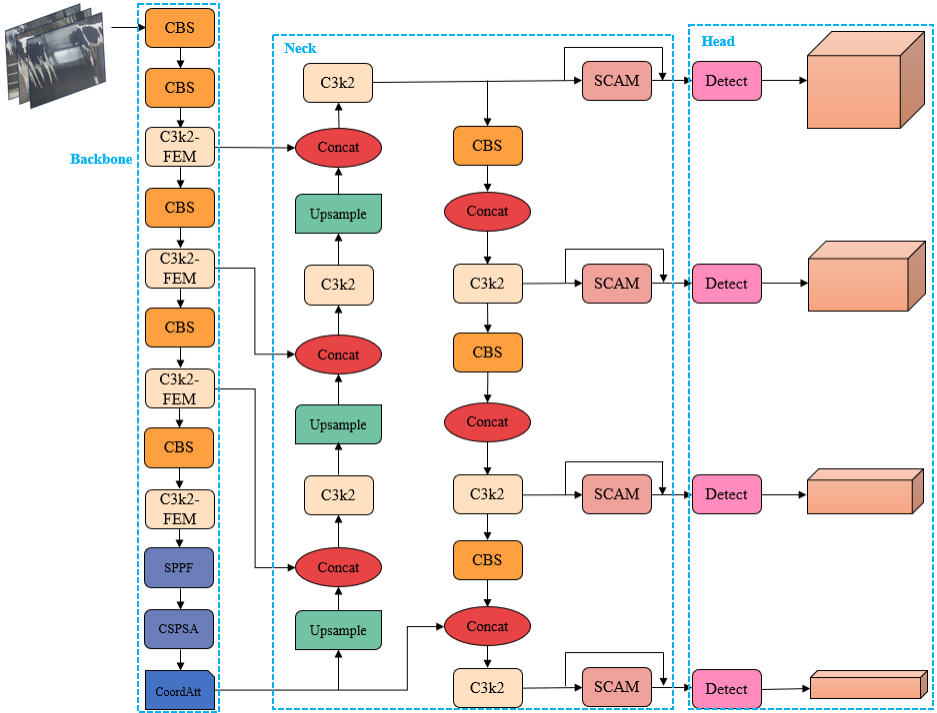
FSCA-YOLO network architecture diagram.

After optimization, the FSCA-YOLO (Feature-enhanced Spatial-Channel Attention YOLO) network structure is as shown in Figure 11. In the backbone, the original C2f module is replaced by the C3k2-FEM module, where the multi-branch and dilated convolution design in FEM enhances feature representation, accommodating the complexity of dairy cow behaviors. In addition, the CoordAtt module is introduced at the end of the backbone to adaptively emphasize behavior-related key features and enrich spatial information modeling. In the neck, the SCAM module is employed to filter the features fed into the detection head, retaining only the most relevant information and dynamically adjusting the attention distribution. This improves detection performance under complex farming environments. Furthermore, part of the features are forwarded to the detection head via residual connections, which increases the flexibility of feature transmission and enhances the model’s generalization capability.

### 2.3. Experimental Environment and Parameter Settings

Table 1 lists the computer hardware environment used for the experiments. During the model training process, the performance of the model is closely related to certain hyperparameter settings.

Selecting appropriate hyperparameters plays a crucial role in enhancing model performance as shown in Table 2.

### 2.4. Evaluation Metrics

To assess the effectiveness of the proposed cow behavior recognition model, this study employs precision, recall, and mean average precision (*mAP*) as evaluation indicators. A predicted bounding box is considered a true positive if its Intersection over Union (*IoU*) with the ground truth exceeds a predefined threshold; otherwise, it is treated as a false positive or false negative, depending on the context. Specifically, True Positives (*TP*) denote correctly identified positive instances, False Positives (*FP*) refer to incorrect positive detections, and False Negatives (*FN*) represent missed detections. The average precision (*AP*) is defined as the area under the precision–recall curve for an individual class, while *mAP* is calculated by averaging *AP* values across all *k* behavior categories:

(11) $$\begin{array}{cc} P & =\frac{TP}{TP+FP} \end{array}$$

(12) $$\begin{array}{cc} R & =\frac{TP}{TP+FN} \end{array}$$

(13) $$\begin{array}{cc} AP & =\int_{0}^{1}PR\;dr \end{array}$$

(14) $$\begin{array}{cc} mAP & =\frac{\sum_{i=1}^{k}AP_{i}}{k} \end{array}$$

## 3. Results

### 3.1. Loss Function Comparison

Based on the initial YOLOv11 backbone network, several commonly used loss functions mentioned above—including Generalized Intersection over Union (GIoU), CIoU, and DIoU—were evaluated, along with the SIoU loss function adopted in this study. The analysis results are shown in Table 3.

**Figure 12:**
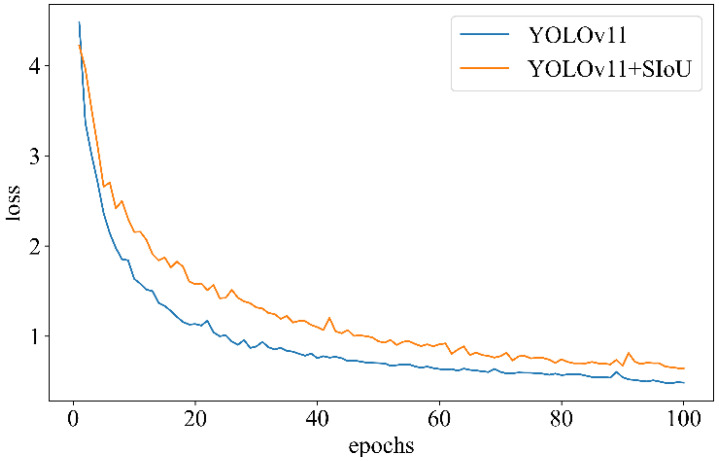
Comparison curve of loss functions. Training loss vs. epoch for YOLOv11 (blue line, baseline) and YOLOv11 + SIoU (orange line). Note: because the SIoU-based objective has a different formulation and numerical scale from the IoU-family losses, the absolute loss values across curves are not directly comparable. Model comparison is therefore based on the validation metrics, where SIoU achieves higher precision, recall, and mAP (see Table 3).

From the table, it can be seen that compared to the underperforming DIoU loss, the SIoU loss function improves precision and recall by 0.8% and 1%, respectively. This improvement is attributed to SIoU and its incorporation of angle as a correction factor when refining the predicted bounding boxes, allowing for better alignment with the actual target boxes. As a result, the model’s accuracy in recognizing and classifying cow behaviors is enhanced. Additionally, during training, SIoU provides more precise gradient information, enabling the model to update parameters in a more optimal direction as illustrated in Figure 12. This precise guidance significantly contributes to the training process by accelerating model convergence.

### 3.2. Attention Mechanisms Comparison

To evaluate the performance of different attention mechanisms within the current network, this study incorporates the SE, Convolutional Block Attention Module (CBAM), and CoordAtt attention module into the backbone network for training and comparison. The experimental results are presented in Table 4.

The SE attention mechanism neglects positional information and demonstrates the weakest performance among the three. In contrast, CBAM considers both channel and spatial information, enhancing the representational capacity of feature maps. As a result, integrating CBAM into the backbone network can improve its performance, though the improvement is not substantial. CoordAtt captures long-range dependencies in one spatial direction while preserving spatial information in the other, embedding positional information into channel attention. Compared with CBAM, incorporating CoordAtt into the backbone network results in a 0.4% increase in precision, a 0.3% increase in recall, and a 0.3% improvement in mAP. These findings are consistent with those reported by Zheng et al. [38].

### 3.3. Ablation Study

Through ablation experiments on each module, the proposed improvement strategies were shown to have a positive impact on the baseline YOLOv11 network. However, the performance improvements varied depending on the specific strategy added. As shown in Table 5, incorporating the FEM-SCAM module improved feature extraction capabilities, leading to a 0.5% increase in precision, a 0.8% increase in recall, and a 0.7% increase in mAP. The introduction of the CoordAtt attention mechanism resulted in a 0.2% increase in precision, a 0.4% increase in recall, and a 0.4% increase in mAP. This suggests that CoordAtt enhances the model’s ability to analyze both channel and spatial features, thereby improving overall performance. Adding the SIoU module further increased the mAP by 0.6%. Finally, compared to the model without an additional detection head, incorporating a small-object detection head led to a 0.5% improvement in precision, a 0.3% increase in recall, and a 0.4% boost in mAP. This indicates that even after feature extraction capabilities have reached a bottleneck, focusing on small-object detection can still further enhance model performance.

### 3.4. Comparative Experiment

To evaluate the performance differences between the improved recognition model and other mainstream object detection models, this section selects several classic models, including Single-Shot MultiBox Detector (SSD) [53], Faster R-CNN [54], YOLOv5, YOLOv8, and YOLOv11, and conducts a comprehensive comparison with the improved YOLOv11 model as shown in Table 6. All experiments—including the benchmark models in Table 6—used the same 3360-frame video dataset collected from livestock farm. According to the data in the table, the FSCA-YOLO model outperforms the other models in terms of precision, recall, and mAP on the collected cow behavior dataset. While SSD shows better performance than Faster R-CNN, the FSCA-YOLO model achieves 5.4% higher precision, 4.3% higher recall, and 4.2% higher mAP compared to SSD.

Within the YOLO series, newer models such as YOLOv8 and YOLOv11 are selected for comparison. The experimental results indicate that YOLOv11 performs better than other YOLO variants. However, compared with the FSCA-YOLO model, YOLOv11 still lags behind by 1.6% in precision, 1.8% in recall, and 2.1% in mAP. Therefore, the FSCA-YOLO model is better suited for achieving more accurate recognition of cow behaviors.

### 3.5. Visualization Results and Analysis

**Figure 13:**
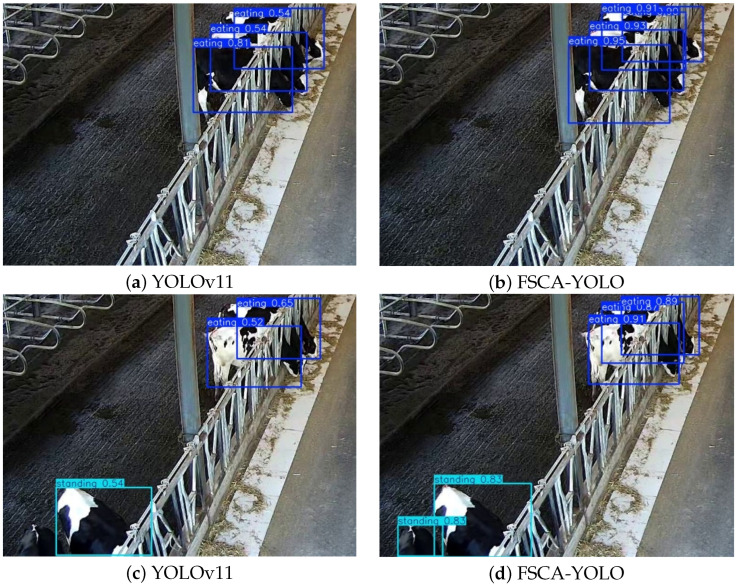
Feeding behavior recognition: YOLOv11 vs. FSCA-YOLO.

To further validate the model’s performance in recognizing different cow behaviors, this section selects cow behavior images from various scenarios. As shown in Figure 13, cows often appear in overlapping positions while feeding. Due to the feeding posture and the structure of the feed trough, occlusions frequently occur in the images to be recognized, posing challenges for behavior recognition. The baseline YOLOv11 model has difficulty correctly identifying feeding behavior when cows are located at the far end of the camera’s field of view and partially occluded. For example, in Figure 13a, two cows are mistakenly detected as one. In addition, some cows have coat patterns that resemble the background environment. As illustrated in Figure 13c, the cow in the lower left corner is missed due to its tail blending with the dark floor. In contrast, as shown in Figure 13b,d, the FSCA-YOLO model—enhanced by modules such as FEM-SCAM, which improve feature extraction capability—demonstrates better discrimination between overlapping cows and between cows and the background, even within limited image capture areas. This ensures the integrity and accuracy of cow behavior recognition.

**Figure 14:**
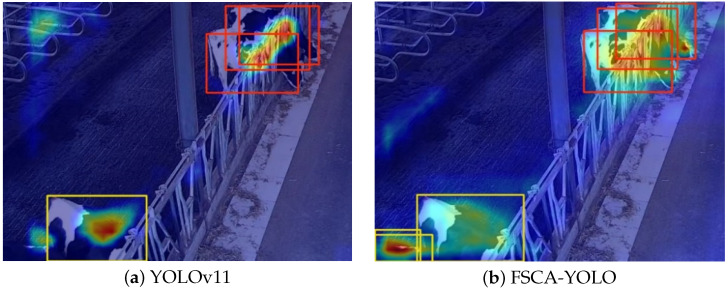
Feature extraction heatmap: YOLOv11 vs. FSCA-YOLO.

In the process of image recognition, whether the model’s attention aligns with the actual position of the target greatly affects its recognition performance. Heatmaps provide an intuitive way to visualize the regions the model focuses on. In a heatmap, darker colors indicate areas where the model’s feature extraction is more concentrated. To better understand the differences in feature extraction between models in cow feeding scenarios, this section compares the baseline YOLOv11 model with the proposed FSCA-YOLO model as illustrated in Figure 14.

As shown in Figure 14a, there are no cows lying in the resting area, and the current recognition task focuses on feeding behavior. However, the baseline YOLOv11 model performs some feature extraction in the upper left corner of the image, indicating that the model fails to concentrate on the key regions. Additionally, for the missed cow in the lower left corner, the attention from YOLOv11 is significantly weaker than that in Figure 14b, leading to a missed detection of feeding behavior. In contrast, the heatmap of the FSCA-YOLO model shows that its attention for the clustered feeding cows is not limited to the shoulder area. Instead, it focuses on the entire body, especially on distinctive features such as the torso and head. This broader feature extraction coverage allows the model to better handle occlusions and avoids unnecessary attention to irrelevant regions, thereby improving recognition efficiency. Moreover, in the previously missed lower-left region, the improved YOLOv11 model allocates more attention, effectively addressing the issue of missed detections.

**Figure 15:**
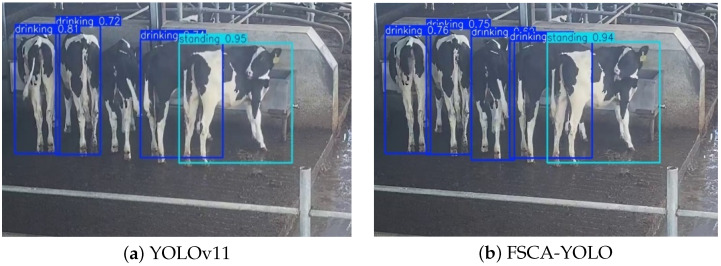
Drinking behavior recognition: YOLOv11 vs. FSCA-YOLO.

When cows are drinking, they often face away from the camera, making the effective feature region for this behavior the smallest among the four behavior types. When the baseline YOLOv11 is used to recognize the drinking area, the continuous and tightly clustered posture of the cows may lead to missed detections as shown in Figure 15. In contrast, the FSCA-YOLO model successfully identifies all drinking behaviors, demonstrating its ability to extract more useful behavior information from limited visual features.

**Figure 16:**
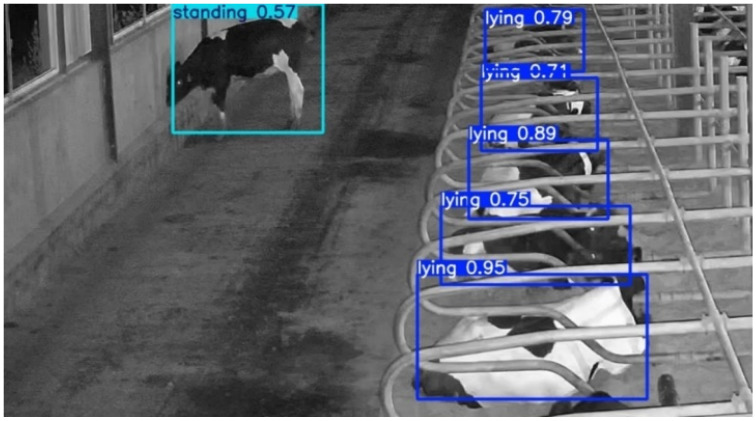
FSCA-YOLO infrared image recognition results.

At night, when lighting is insufficient, the camera automatically switches to infrared mode. Compared to daytime images, infrared images capture fewer details such as the patterns and colors on the cow’s body. To evaluate whether FSCA-YOLO can effectively recognize cow lying behavior under nighttime conditions, this section further investigates its performance on infrared images. As shown in Figure 16, when cows are lying down, their bodies are often partially obscured by the bedding, which further increases the difficulty of recognition. Experimental results demonstrate that FSCA-YOLO is still able to accurately detect all cow behaviors in infrared images, confirming that the proposed behavior recognition model is capable of performing well in nighttime conditions. To compare infrared (IR) with daytime performance, this study stratified the held-out test set by lighting condition using the camera’s IR-mode flag and timestamps (daytime vs. night), and applied the same FSCA-YOLO model on both subsets with identical preprocessing, inference thresholds (confidence/Non-Maximum Suppression (NMS)), and evaluation metrics (precision, recall, mAP@0.5, and per-behavior accuracy).

**Figure 17:**
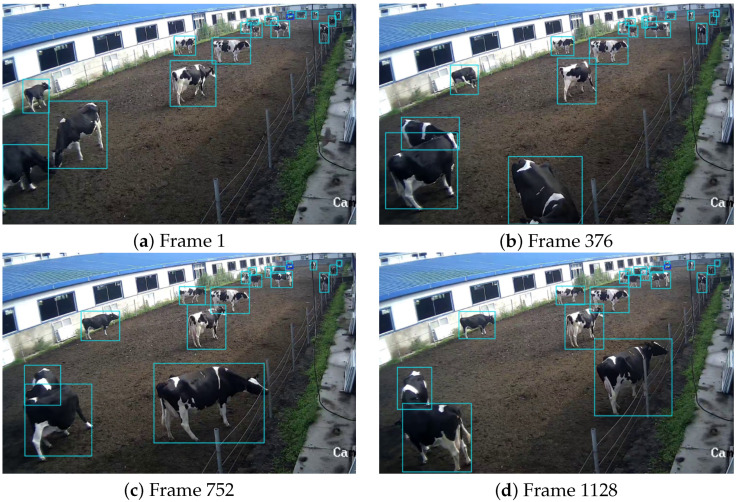
FSCA-YOLO Outdoor scene recognition results. An outdoor paddock with high cow density and long-range small targets. To maintain legibility in this crowded scene, per-detection text overlays (confidence scores and behavior labels) are suppressed in the visualization. The model nevertheless outputs scores and labels for every detection, which were used for quantitative evaluation; the omission here affects visualization only.

Compared to indoor areas, outdoor cow activity areas are more spacious, resulting in the frequent appearance of small-target cows in outdoor images. Additionally, during peak activity periods, the large number of cows outdoors poses a challenge for multi-object detection. In this section, FSCA-YOLO is used to recognize cow behaviors in outdoor scenarios, and the results are shown in Figure 17. Because the figure depicts a crowded outdoor scene with many cows—including small, distant targets—overlaying text for every detection would obscure the boxes and hinder error inspection. We therefore suppress the per-detection behavior labels and confidence scores in the visualization to maintain readability. Cows located at the far end of the camera’s field of view appear smaller in size, but thanks to the inclusion of the P2 small-object detection layer, the model can accurately identify these small targets. Experimental results demonstrate that FSCA-YOLO meets the recognition requirements for cow behavior in outdoor activity areas.

### 3.6. Cow Counting via FSCA-YOLO

Accurate regional cow counting supports both group behavior analysis and health management. On one hand, variations in cow numbers reflect group behavior trends [55]; on the other, overcrowding may lead to heat stress, especially under high temperatures due to poor ventilation, requiring timely intervention [56]. Moreover, analyzing behavior differences under varying group sizes—such as feeding patterns—can inform more efficient feeding strategies through combined analysis of cow count and feed consumption [57]. Regional cow counting is one of the key methods to ensure the proper functioning of the overall system.

**Figure 18:**
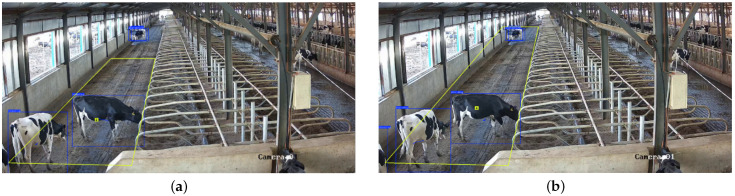
Effect of cow counting under different detection region sizes. (**a**) Initial frame with the default Region-of-interest (ROI) and detection overlays. (**b**) ROI after mouse-drag refinement. The yellow quadrilateral denotes the user-defined recognition region; blue boxes indicate detected cows; the numeric overlay inside the ROI shows the number of cows classified as Standing within the ROI. Areas outside the ROI (e.g., the feeding area on the right) are ignored to reduce computation and avoid irrelevant detections.

In this section, OpenCV is used to calibrate the target recognition region and to visualize it by drawing the designated area on the image display interface. First, a coordinate acquisition tool developed using OpenCV is employed to obtain the coordinates of detection regions in video frames. Then, to enhance the user experience in observing relevant data, a mouse-drag module is developed using OpenCV. With this module, users can flexibly adjust the detection boxes based on actual needs by dragging with the mouse. In terms of detection region configuration, a hyperparameter interface is designed to allow easy adjustment of detection parameters in future applications to meet requirements of different scenarios. Ultimately, the recognition results are intuitive and clear as shown in Figure 18. The yellow quadrilateral outlines the defined detection area, and the number displayed within indicates the number of standing cows identified in that region by the behavior recognition model. By precisely delineating the detection area, the system avoids unnecessary computational costs from processing irrelevant areas. For example, cows in the feeding area on the right side of the image are excluded from analysis because they fall outside the defined region, thus improving model efficiency and ensuring that the recognition results are more accurate and meaningful.

## 4. Discussion

In this study, multiple improvements are introduced to the original YOLOv11 model to enhance its performance in complex dairy farming environments. In terms of model architecture, the collaborative design of FEM + SCAM and CoordAtt establishes a feature extraction pipeline that simultaneously enhances spatial and channel information. At the task level, the added detection head improves the model’s sensitivity to small targets, enabling the better recognition of small-scale cows or partially occluded individuals in group scenarios. At the application level, this study integrates the FSCA-YOLO model seamlessly with an OpenCV-based zone counting and tracking toolkit, which substantially improves the overall system performance and data quality. The proposed improvements to the YOLOv11 model provide a reliable and efficient vision-based solution for the real-time, multi-object behavior recognition of dairy cows across various environmental settings.

Despite the effectiveness of the proposed system in accurately recognizing and counting cow behaviors within specific regions, there remain areas that require further improvement due to limitations in equipment and environmental conditions. In particular, the model occasionally misclassifies eating cows as standing when the head and neck are partially occluded by the feeder or by other cows. In addition, in crowded scenes with high inter-cow overlap, detection confidence may decrease, leading to occasional missed or false identifications. These cases highlight the challenges of distinguishing subtle behavior differences under occlusion and suggest that further enhancement of fine-grained feature extraction and temporal context modeling will be necessary in future work. These limitations provide directions for future research and system enhancement as discussed below.

### 4.1. Monocular Camera Depth Limitations

In typical farm environments, surveillance systems predominantly rely on monocular cameras, which cannot directly capture depth data [58]. As a result, it is currently difficult to obtain precise measurements of cow movement distances, particularly for behaviors such as standing and walking [59,60]. Future work may focus on estimating walking distances using monocular vision techniques—such as structure-from-motion (SfM), monocular depth estimation based on deep learning, or geometric projection methods—to enrich behavior analysis with spatiotemporal movement data [61,62].

### 4.2. Behavior Tracking and Annotation Challenges

This study primarily focuses on group-level behavioral patterns [63] within designated regions, while paying relatively less attention to individual cows. However, tracking and analyzing individual behavior over time is crucial for fine-grained livestock management [64]. Future research should consider integrating computer vision techniques for individual cow identification [65,66] (e.g., based on visual features, tags, or biometric patterns), enabling cross-region identity tracking and the establishment of behavioral profiles [63] for each cow. This would significantly enhance the precision and personalization of farm management practices, laying a foundation for data-driven precision livestock farming. To achieve identity-consistent analysis in group-housed environments, future work may extend this framework to multi-object behavior tracking by coupling the detector with a tracking pipeline and evaluating performance using Multiple Object Tracking Accuracy (MOTA), Multiple Object Tracking Precision (MOTP), Identification F1 Score (IDF1), and Higher-Order Tracking Accuracy (HOTA) metrics [28].

In addition, behavioral annotation, especially in crowded scenes, remains a subjective and error-prone process [67]. Some behaviors, such as “standing idle” vs. “standing alert”, may have ambiguous boundaries. To mitigate this issue, future studies could incorporate multiple annotators, consensus mechanisms, or even semi-supervised learning approaches to improve label quality and reduce human bias [68,69]. The realization of data-driven, precise, and personalized livestock management is contingent upon the simultaneous achievement of individual identity traceability and high-confidence behavior annotation.

### 4.3. Potential of Multimodal Sensing Integration

Current dairy cow behavior recognition systems primarily rely on visual data [70], which, despite being rich and non-invasive, are susceptible to occlusions [71], lighting changes, and limited viewpoints. To address these shortcomings, integrating complementary sensing modalities offers a promising direction. Inertial measurement units (IMUs) can capture subtle motion patterns; Radio Frequency Identification (RFID) enables reliable individual identification and positioning; environmental sensors provide contextual data related to stress and behavior; and acoustic sensors detect vocalizations linked to feeding, estrus, or distress [72]. Multimodal data fusion enables more robust and accurate behavior recognition, supports early disease detection and welfare monitoring [72], and facilitates intelligent, data-driven livestock management. Such integration is key to advancing precision dairy farming toward greater adaptability and resilience.

Overall, the findings of this study not only demonstrate the feasibility of applying enhanced deep learning models for behavior recognition in real farming contexts but also highlight critical directions for future system upgrades. By addressing current limitations—including depth perception, individual-level tracking, annotation ambiguity, and sensor fusion—future research can push the boundaries of intelligent livestock monitoring systems. In particular, improving the reliability of behavior labeling through consensus annotation or semi-supervised methods will enhance model generalizability, while integrating multimodal sensing (e.g., inertial, acoustic, and environmental) will provide a richer context for behavior and health interpretation. Beyond technical aspects, effective dissemination is also essential. Social media platforms, such as Instagram, have been shown to convey complex scientific topics to non-specialist audiences [73]. Leveraging such channels could help raise awareness of automated cow behavior recognition and its relevance for animal welfare and farm management. Ultimately, these advancements will contribute to the realization of adaptive, data-driven, and ethically sound precision livestock farming, promoting not only animal welfare and health but also operational efficiency and sustainability.

## 5. Conclusions

In this work, several improvements are made to the original YOLOv11-based cow behavior recognition model. The FEM-SCAM module enhances feature extraction and suppresses irrelevant information, while the CoordAtt attention mechanism improves the capture of positional and fine-grained details. A small-object detection head is added to better detect cows at the far end of the camera view, and the SIoU loss function is introduced to accelerate convergence and improve detection accuracy. On the self-built cow behavior dataset, which denotes our custom-annotated set of 3,360 barn images with per-cow bounding boxes and four behavior labels (standing, feeding, drinking, and lying), all images were collected from indoor and outdoor barn videos under both day and night conditions, for which FSCA-YOLO demonstrates exceptional recognition performance. Compared with existing models, it significantly reduces missed detections while maintaining a remarkably low false positive rate, leading to a notable improvement in overall detection robustness. FSCA-YOLO excels particularly in challenging scenarios involving cow occlusion, limited feature information, or high similarity between cow features and the background. It significantly reduces missed and false detections, meeting the demands for accurate recognition of cow behaviors in various regions. Moreover, the model benefits from enhanced contextual awareness and adaptive attention mechanisms, which further improve its ability to distinguish fine-grained behaviors and adapt to dynamic scenes with varying lighting and background interference.

Experimental results show that the enhanced model achieves superior performance compared to mainstream detectors, with a precision of 95.7%, recall of 92.1%, and mAP of 94.5%. It effectively handles occlusions in drinking and feeding scenarios, accurately identifies behaviors under low-light infrared conditions, and maintains robust performance in outdoor multi-object environments. To enable flexible behavior recognition and region-based cow counting, OpenCV is integrated with the model, supporting diverse application needs in multi-target behavior tracking systems.

FSCA-YOLO demonstrates significant improvements over existing methods on our self-built dataset; however, its generalization ability still requires validation on larger-scale and multi-farm datasets. In future work, the model should be validated across a wider range of farming conditions. This includes testing on different cow breeds to ensure breed-independent performance, extending behavior categories beyond those considered in this study, and evaluating robustness under diverse environmental settings such as varying lighting, weather, and barn structures. Such studies will be essential to fully establish the generalizability and practical value of FSCA-YOLO in precision livestock farming.

## Figures and Tables

**Table 1:** Computer hardware environment.

Configuration Item	Parameter Value
CPU	Intel(R) Xeon(R) Gold 5218R
GPU	GeForce RTX 2080 Ti
Memory	94 GB
Operating System	Ubuntu 16.04
Development Environment	Python 3.9
Accelerated Environment	CUDA 11.1

**Table 2:** FSCA-YOLO training parameters.

Hyperparameter	Value
Optimization	SGD
Initial Learning Rate	0.01629
Momentum	0.98
Weight Decay	$4.5\times 10^{-4}$
Batch Size	8
Epochs	100

**Table 3:** Performance metrics of different loss functions.

Loss Function	Precision (%)	Recall (%)	mAP (%)
DIoU	93.5	90.8	91.5
GIoU	93.9	91.2	92.1
CIoU	94.1	90.3	92.4
SIoU	94.3	91.8	93.1

**Table 4:** Performance metrics of different attention mechanisms.

Attention Model	Precision (%)	Recall (%)	mAP (%)
SE	93.8	90.6	92.4
CBAM	94.2	90.8	92.8
CoordAtt	94.6	91.1	93.1

**Table 5:** Performance metrics after adding different modules to the model.

FEM-SCAM	CoordAtt	SIoU	4Head	Precision (%)	Recall (%)	mAP (%)
				94.1	90.3	92.4
✓				94.6	91.1	93.1
✓	✓			94.8	91.5	93.5
✓	✓	✓		95.2	91.9	94.1
✓	✓	✓	✓	95.7	92.1	94.5

**Table 6:** Performance metrics of different detection models on the validation set.

Model	Precision (%)	Recall (%)	mAP (%)
Faster R-CNN	90.2	87.0	87.1
SSD	90.3	87.8	90.3
YOLOv5	92.2	86.2	91.9
YOLOv8	92.8	88.1	90.2
YOLOv11	94.1	90.3	92.4
FSCA-YOLO	95.7	92.1	94.5

## Data Availability

The datasets generated, used, and/or analyzed during the current study will be available from the corresponding author upon reasonable request.
